# Genetic Analysis of Methane Emission Traits in Holstein Dairy Cattle

**DOI:** 10.3390/ani13081308

**Published:** 2023-04-11

**Authors:** Stephanie Kamalanathan, Kerry Houlahan, Filippo Miglior, Tatiane C. S. Chud, Dave J. Seymour, Dagnachew Hailemariam, Graham Plastow, Hinayah R. de Oliveira, Christine F. Baes, Flavio S. Schenkel

**Affiliations:** 1 Centre for Genetic Improvement of Livestock, Department of Animal Biosciences, University of Guelph, Guelph, ON N1G 2W1, Canada; 2 Lactanet Canada, Guelph, ON N1K 1E5, Canada; 3 Centre for Nutrition Modelling, Department of Animal Biosciences, University of Guelph, Guelph, ON N1G 2W1, Canada; 4 Department of Agricultural, Food & Nutritional Science, University of Alberta, Edmonton, AB T6G 2P5, Canada; 5 Institute of Genetics, Vetsuisse Faculty, University of Bern, Bremgartenstr. 109a, 3012 Bern, Switzerland

**Keywords:** methane production, methane intensity, methane yield, genetic parameters

## Abstract

**Simple Summary:**

Dairy cows contribute to greenhouse gas emissions from livestock, and reducing methane emissions is vital for the long-term sustainability of the dairy industry. Genetics and breeding strategies can be used to bring about permanent and long-term enteric methane emission reduction from dairy cattle. Here, we assess three definitions for methane emission traits, investigate their genetic parameters, and compare potential implications of including them in a genetic selection program. All three commonly used methane traits (daily methane production, methane yield, and methane intensity) were heritable, and are potential candidates for a selection program. Additionally, all traits were highly correlated with each other, indicating that selection on one trait would lead to an indirect response on the other methane traits. By exploring trait definitions for including methane in selection strategies, this work contributes to potential mitigation strategies for reducing greenhouse gas emissions in dairy cattle using genetics.

**Abstract:**

Genetic selection can be a feasible method to help mitigate enteric methane emissions from dairy cattle, as methane emission-related traits are heritable and genetic gains are persistent and cumulative over time. The objective of this study was to estimate heritability of methane emission phenotypes and the genetic and phenotypic correlations between them in Holstein cattle. We used 1765 individual records of methane emission obtained from 330 Holstein cattle from two Canadian herds. Methane emissions were measured using the GreenFeed system, and three methane traits were analyzed: the amount of daily methane produced (g/d), methane yield (g methane/kg dry matter intake), and methane intensity (g methane/kg milk). Genetic parameters were estimated using univariate and bivariate repeatability animal models. Heritability estimates (±SE) of 0.16 (±0.10), 0.27 (±0.12), and 0.21 (±0.14) were obtained for daily methane production, methane yield, and methane intensity, respectively. A high genetic correlation (rg = 0.94 ± 0.23) between daily methane production and methane intensity indicates that selecting for daily methane production would result in lower methane per unit of milk produced. This study provides preliminary estimates of genetic parameters for methane emission traits, suggesting that there is potential to mitigate methane emission in Holstein cattle through genetic selection.

## 1. Introduction

The global population is rapidly increasing, and environmentally sustainable food production is becoming a research priority. The agricultural industry is often targeted for its contribution to climate change due to livestock production. Although dairy cattle represent only a fraction of total greenhouse gas (GHG) emissions, increased awareness of their contribution to the problem has resulted in consumer pressure on the dairy industry to improve its efficiency and sustainability and to investigate ways to mitigate its environmental footprint. Methane is the primary GHG emitted by ruminant livestock, and is generated by the normal feed digestion process; considering that methane is considered to be one of the more potent GHGs, its mitigation is of high priority to the dairy cattle industry.

Several systems for measuring direct methane production have become available, including respiration chambers, the automated head-chamber system (e.g., GreenFeed system; C-Lock, Inc., Rapid City, SD, USA), and the sulfur hexafluoride (SF6) tracer gas system (e.g., SF6 Tracer Technique Guidelines [1]). Studies comparing these three systems have concluded that daily methane production measured via GreenFeed systems is similar to that obtained using SF6 and respiration chambers [2,3]. Research groups in Spain have put considerable effort into comparing methods of recording methane emissions and implementation strategies [4,5]. Finally, the International Committee for Animal Recording (ICAR), through the Feed and Gas working group, has outlined guidelines for collecting methane emissions data, which is imperative for future genetic evaluation of the trait [6]. Although today’s average dairy cow produces 20% more GHG than her counterpart in the 1990s, she produces 46% more milk [7], meaning that fewer cows are required to maintain production. The overall number of cows has decreased by 31% through improved genetics and changes in feeding and management practices since the 1990s, ultimately reducing the emissions associated with dairy cows by 17% [7]. Nevertheless, the voluntary feed intake of these animals, which is a key driving force of methane emissions, has increased as well [8,9]. Therefore, focusing on ways to improve individual animal emissions is a priority.

Previous studies have shown that methane emissions from dairy cattle have a heritable component that ranges from 0.12 to 0.45 depending on the trait analyzed and the stage of lactation (e.g., methane intensity, defined as g of methane emitted over a week per fat and protein corrected milk, and early-to-middle or late lactation) [10,11,12]. As methane emission is heritable, genetic selection is a viable option that may lead to cumulative long-term improvement. That said, there is no consensus on the best methane-related trait to consider in breeding programs. Therefore, the objective of this study was to investigate potential methane emission traits (methane production, methane yield, and methane intensity) with respect to their heritability and subsequent ability to rank selection candidates.

## 2. Materials and Methods

### 2.1. Ethics and Animal Care

Animal Care Committee approval was obtained from the University of Guelph (animal utilization protocol number: 3503) and from the University of Alberta (animal utilization protocol number: AUP00000170).

### 2.2. Data Collection

The data used in this study were collected through the Efficient Dairy Genome Project (https://genomedairy.ualberta.ca (accessed on 7 April 2023)), a global initiative investigating the use of selective breeding and novel genomics technologies to improve feed efficiency and reduce methane emissions in dairy cattle. Data from two Canadian research stations located in Alberta and Ontario were used in the analyses. The data collection in these two stations are described below.

#### 2.2.1. Ontario Dairy Research Centre

For the portion of the study conducted at the Ontario Dairy Research Centre (ODRC; Elora, ON, Canada) at the University of Guelph’s dairy research facility, first lactation cows between 120 and 150 days in milk (DIM) were moved in groups of two to four into a tie-stall area of the barn for methane emission testing. During their time in the tie-stall, animals were fed a total mixed ration (TMR) ad libitum delivered daily at approximately 11:00 h and had ad libitum access to water. The average ingredient composition of the feed was corn silage (30%), haylage (28%), high-moisture corn (26%), supplement (12%), and chopped straw (2%). A detailed composition analysis is available in Seymour et al. [13]. At each test time, all feed was removed from the manger and the GreenFeed equipment was moved in front of the animal using a conventional pallet jack. Methane emissions were measured for approximately 10 min, after which the equipment was moved away from the animal and allowed to recalibrate for 3 min before testing the next animal. After each testing at 12:00 h and 16:00 h, approximately 25% of the daily allotment of feed was added back to the manger, with the remaining feed added after the 20:00 h test time. Cows were milked twice daily in their stalls at 05:30 h and 17:30 h. Cows were habituated to the barn and testing protocol for three days before being tested for five consecutive days using a GreenFeed system (GreenFeed; C-Lock Inc., Rapid City, SD, USA). The GreenFeed system was calibrated as per Gerrits et al. [14]. Cows were tested four times a day at 08:00 h, 12:00 h, 16:00 h, and 20:00 h. A testing session started when the researcher pushed the GreenFeed toward the cow and the cow entered her head in the semi-enclosed head hood. Infrared sensors in the GreenFeed detected the position of the cow’s head and its RFID tag for individual identification. Each testing session lasted approximately 10–12 min to ensure sufficient time during which the cow’s head was in the proper position and breath samples could be collected to estimate methane and carbon dioxide flux based on airflow and methane and carbon dioxide concentrations. The concentration of gases was corrected for background concentrations and adjusted to standardized temperature, humidity, and pressure [6]. Automated feed drops (every 23 s) of pellets from the overhead hopper were used to motivate the cow to keep her head in the machine for the duration of testing. When needed, the testing period was prolonged to make sure the cow had her head in the machine for a minimum of 10 min. Methane data in g/day estimated by the GreenFeed system were extracted for each cow’s testing session during a day over the course of the five testing days. Therefore, methane production on each day was the average of the estimated methane production during the testing sessions (8:00, 12:00, 16:00, and 20:00) provided by the GreenFeed system (in g/day).

#### 2.2.2. Dairy Research and Technology Center

The Dairy Research and Technology Center (DRTC) is part of the University of Alberta infrastructure. Mixed parity cows between 30 DIM and 250 DIM were housed in a ventilated tie-stall barn with ad libitum access to water and TMR, which was delivered daily at approximately 08:00 h. The average ingredient composition of the feed was barley silage (58%), alfalfa hay (10%), and supplement (31%). Cows were milked twice per day at 03:00 h and 15:00 h. Animals had access to an exercise area (an open dry lot) for 3 h every second day. Methane measurements were collected using the GreenFeed system from 2016 to 2020. Measurements were collected on groups of 10–15 mixed parity lactating cows. Groups were measured over twelve consecutive days at 12-h intervals, providing two measurements per day. The first day measurement was conducted at 01:00 h and 13:00 h, then shifted by one hour every day to cover all 24 h throughout the twelve days of recording. In 2019, this method was adjusted to correspond more closely to the ODRC herd, where measurements were made at three time points per day (08:00 h, 12:00 h, and 16:00 h) for five consecutive days. The GreenFeed system was adjusted so that each cow could receive drops (approximately every 40 s) of the barley grain used to bait the cow to the head compartment. During the entire CH_4_ emission measurement period, CO_2_ recovery tests were performed at the start of each group and gas calibration was performed every week during the experimental period. The details of the methane emission measurement methods have been described in our previous study [15].

### 2.3. Variation in Methane Testing

Potential differences in methane emission among recording times (08:00 h, 12:00 h, 16:00 h, and 20:00 h) were tested for data collected at ODRC using R Version 3.5.0 software [16] via the VAR.TEST and T.TEST functions. F-tests and a two-sided Welch’s *t*-test, which assumes unequal variances between the groups, were conducted to test the differences in variances and means between the different measurement time periods, respectively. This was done to evaluate whether there were specific time points at which methane emission peaked or variation in methane outputs corresponding to the time of measurement. Differences were considered significant at 5% probability.

### 2.4. Data Set and Methane Traits

A total of 2469 methane emission records were collected on 422 Holstein cows from August 2016 to March 2020 from the two research herds. Only first lactation cows were analyzed. Records ranged from 26 to 257 DIM from cows who calved between 22 and 33 months of age. Possible outliers were removed using a 1.5 × interquartile range. Cows were required to have at least three methane measurements (days) for analysis, as methane measurements from both herds were collected more than once per cow to assess variability. The pedigree file used for all methane traits in the analysis included 1434 animals, consisting of 892 dams and 253 sires over 25 generations.

Various methane emission traits are used in livestock species [17,18]. This study focused on three main methane traits: methane production (MeP; total grams of methane produced per day), methane yield (MeY; grams of methane produced per kg of dry matter intake per day), and methane intensity (MeI; grams of methane produced per kg of milk per day).

### 2.5. Variance Components

Variance components were estimated using the Average Information Restricted Maximum Likelihood (AIREML) methodology and a repeatability animal model for MeP, MeY, and MeI. All analyses were performed using the ASReml software Version 4.1 [19]. Connectedness between contemporary groups (in this study, herd-year-season or herd-year, depending on the trait) were verified using the AMC program [20], which takes into account the number of genetic links between contemporary groups (CGs). Up to two CGs were disconnected depending on the methane trait and were removed from the dataset. The univariate repeatability animal model and the variance–covariance structure used are described below:

$$y_{ijklmn}=\mu +{ADC}_{i}+{CG}_{j}+{DIM}_{k}+a_{l}+{pe}_{m}+e_{ijklmn}$$

$$V\left[\begin{array}{c} a\\ p\\ e \end{array}\right]=\left[\begin{array}{ccc} A\sigma_{a}^{2} & 0 & 0\\ 0 & I\sigma_{pe}^{2} & 0\\ 0 & 0 & I\sigma_{e}^{2} \end{array}\right]$$

where $y_{ijklmn}$ is the phenotype for MeP, MeY, or MeI; *μ* is the intercept; ${ADC}_{i}$ is the fixed effect of the *i*th age of cow at calving class (six classes; age ≤ 23, 24, 25–26, 27–28, 29–30, and ≥31 months to balance the number of records per class); ${CG}_{j}$ is the fixed effect of the *j*th herd-year-season of recording (ten classes: MeI; twelve classes: MeP), where season is split in two classes (March–August and September–February) or of the *j*th herd-year of recording (six classes: MeY); ${DIM}_{k}$ is the fixed effect of the *k*th days in milk class (three classes); $a_{l}$ is the random additive genetic effect of the *l*th animal, distributed as $N(0,A\sigma_{a}^{2})$, in which A is the numerator-relationship matrix and $\sigma_{a}^{2}$ is the additive genetic variance; ${pe}_{m}$ is the random permanent environmental effect of the *m*th animal with records, distributed as $N(0,I\sigma_{pe}^{2})$, in which I is an identity matrix and $\sigma_{pe}^{2}$ is the permanent environmental variance; and $e_{ijklmn}$ is the random residual effect of the *n*th observation.

A better and less restrictive model for repeated records, such as a random regression model, was not used because of the limitations imposed by the sample size.

Bivariate models were used to estimate genetic, permanent environment, and phenotypic correlations using the same effects as in the univariate model. Phenotypic correlations were estimated as $r_{p}=\frac{\sigma_{pj,pk}}{\sqrt{\sigma_{pj}^{2}\sigma_{pk}^{2}}}$, where $\sigma_{pj,pk}$ is the phenotypic covariance between trait *j* and trait *k* and $\sigma_{pj}^{2}$ and $\sigma_{pk}^{2}$ are the phenotypic variance estimates for trait *j* and trait *k*, respectively. Similarly, genetic correlations were estimated as $r_{g}=\frac{\sigma_{aj,ak}}{\sqrt{\sigma_{aj}^{2}\sigma_{ak}^{2}}}$, where $\sigma_{aj,ak}$ is the additive genetic covariance between trait *j* and trait *k* and $\sigma_{aj}^{2}$ and $\sigma_{ak}^{2}$ are the additive genetic variance estimates for trait *j* and trait *k*, respectively. Lastly, permanent environmental correlations were estimated as $r_{pe}=\frac{\sigma_{pej,pek}}{\sqrt{\sigma_{pej}^{2}\sigma_{pek}^{2}}}$, where $\sigma_{pej,pek}$ is the permanent environmental covariance between trait *j* and trait *k* and $\sigma_{pej}^{2}$ and $\sigma_{pek}^{2}$ are the permanent environmental variance estimates for trait *j* and trait *k*, respectively.

The estimates of heritability (*h*^2^) and repeatability (*r*) from the univariate models were calculated as follows:

$$h^{2}=\frac{\sigma_{a}^{2}}{\sigma_{p}^{2}},\;r=\frac{\sigma_{a}^{2}+\sigma_{pe}^{2}}{\sigma_{p}^{2}}$$

where $\sigma_{a}^{2}$ is the additive genetic variance, $\sigma_{p}^{2}$ is a phenotypic variance, and $\sigma_{pe}^{2}$ is the permanent environmental variance.

Heritability of the average of a different number of methane emission records (from 2 to 5) was obtained following Falconer and MacKay [21]:

$$h_{n}^{2}=\frac{h^{2}\times n}{1+\left(n-1\right)\times r}$$

where $h^{2}$ is the heritability estimated from the univariate model, n is the number of measurements from 2 to 5, and r is the repeatability estimated from the univariate model.

### 2.6. Rank Correlations and Accuracy of Estimated Breeding Values

Estimated breeding value (EBV) rank correlations between methane traits were used to assess potential re-ranking, considering either all animals or only sires with daughters (*n* = 103) with records for the methane traits. The Spearman rank correlations were obtained using the COR.TEST function in R Version 3.5.0 software [16]. In addition, the average accuracy of sires with daughters with records for the respective traits was estimated as follows:

$$accuracy=\sqrt{\left(1-\frac{S^{2}}{\left(1+F\right)\sigma_{a}^{2}}\right)}$$

where, S is the standard error of the EBV, $\sigma_{a}^{2}$ is the population additive genetic variance, and F is the inbreeding coefficient of an animal. Inbreeding coefficients were obtained as 1 minus the diagonal of the relationship matrix using the method proposed by Meuwissen and Luo [22] to estimate the elements of the diagonal.

## 3. Results and Discussion

### 3.1. Descriptive Statistics

Descriptive statistics are presented in Table 1 and Table 2. The means of MeP, MeY, and MeI were 463.5 g/d, 23.5 g/kg DMI, and 14.6 g/kg milk, respectively. The means of the methane traits from the ODRC herd were higher than those from the DRTC herd. This could be related to higher average fat production (+27 g/d), lower milk production (−1.8 Kg/d), and lower DMI (−1.4 kg/d) of ODRC cows compared to DRTC cows (Table 2), as methane production is positively correlated with fat production [23]. ODRC cows were heavier on average (+36 kg) then DRTC cows. The coefficient of variation (CV) of all traits ranged between roughly 20% and 35% across herds. The average daily estimated methane production in this study is marginally higher than other findings in the literature using different methodologies. For example, Grainger et al. [24], who measured methane on a small sample of Australian Holstein cows kept under experimental conditions, indicated an average daily methane emission of 331 g when measured using the SF6 tracer method and 322 g when using respiration chambers. However, Huhtanen et al. [3] and Denninger et al. [25], who used GreenFeed and Holstein dairy cows, showed average emissions of 447 g/d and 426 g/d, respectively. Means for the MeY and MeI traits reported in this study were similar to results found before in the literature [3,11,26]. Niu et al. [26] reported an average methane yield of 21.6 g/kg DMI and methane intensity of 13.5 g/kg milk using an international dataset of various recording methods. Additionally, Huhtanen et al. [3] reported an average methane yield of 21.6 kg/d, and Lassen and Lovendahl [11] reported an average of 8.6 g/L for estimated methane intensity using a Fourier-transform infrared spectroscopy technique during milking in an automated milking system (AMS).

### 3.2. Variation over Time

**Figure 1:**
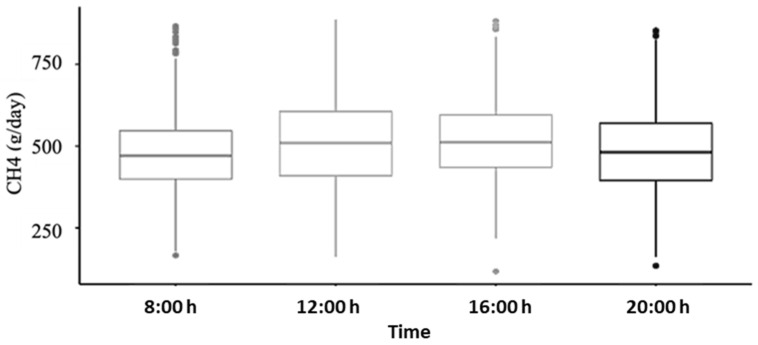
Daily methane production (CH_4_ g/day) from dairy cows fed once daily, measured using a GreenFeed system; the four measurement time points in a day were 08:00 h and 12:00 h, 16:00 h, and 20:00 h. Differences in the means between 12:00 h and 16:00 h and between 20:00 h and 8:00 h were not significant (*p* > 0.05), while differences in variances between 8:00 h and 16:00 h and between 16:00 h and 20:00 h were not significant (*p* > 0.05). All other differences in means and variances were significant (*p* < 0.05). The darker boxplot corresponds to the measurement time that might be eliminated if the number of daily CH_4_ measurements were to be reduced due to the amount of effort required to make all four measurements in one day.

F-tests and Welch two-sample *t*-tests were conducted to test the differences in variances and means between the different recording times (08:00 h and 12:00 h, 16:00 h, and 20:00 h) for the ODRC herd. Differences in the means between 12:00 h and 16:00 h and between 20:00 h and 8:00 h were not significant (*p* > 0.05, Figure 1), while differences in variances between 8:00 h and 16:00 h and between 16:00 h and 20:00 h were not significant (*p* > 0.05). All other differences in means and variances were significant (*p* < 0.05). The results showed that the highest amount of methane is emitted after feeding, increasing by an average of 35 g/cow from 480 g/cow (8:00 h) to 515 g/cow (12:00 h); fresh feed is delivered approximately an hour before the 12:00 h testing. These results agree with the literature. Hristov et al. [27], who used a GreenFeed system to study diurnal methane emissions from dairy cattle fed once daily, found variability between two different time points and increased methane emitted after feeding. In addition, Pszczola et al. [28] observed a pattern of methane emission levels increasing after feeding events and dropping overnight. In the future, adjusting the data for the time of day at which the measurements are recorded should be considered. If measurements are taken at times of the day with a peak or low methane level and the time of measurement is not included in the model, the results could be skewed. The approach in this study was to use the average daily methane emission (g/day) estimated using the four measurements in each day, then average them over the days of measurement to obtain the daily methane emission of a cow, which are the results discussed in the remaining sections. If the number of daily CH_4_ measurements were to be reduced due to the amount of effort to make all four measurements in a day, the night measurement at 20:00 would be a good candidate for elimination, as they have the same mean as the 8:00 measurements and the 8:00 measurements, while the 20:00 measurements have the same variance as the measurements at 16:00.

### 3.3. Variance Component Estimates

Estimated variance components, heritability ($h^{2}$), and repeatability (r) for the traits are presented in Table 3. The estimated heritability and corresponding standard error (SE) for MeP, MeY, and MeI was 0.16 (0.10), 0.27 (0.12), and 0.21 (0.14), respectively. Despite the large standard errors, the heritability estimated for MeP was similar to that previously reported by Breider et al. [10] using random regression in a bivariate analysis with milk yield or body weight (0.15). Additionally, van Engelen et al. [29] estimated heritability for predicted methane production based on milk mid-infrared spectra (0.17). However, other studies have reported more variable estimates for methane production, such as López-Paredes et al. [5], who reported a lower heritability of 0.12 using a nondispersive infrared methane detector to calculate methane production from methane concentrations using an equation derived from Chagunda et al. [30]. Higher methane production heritability estimates ranging from 0.23 to 0.35 using other methodologies have been reported, such as those estimated using direct measurement with the SF6 and predicted measurement methods [11,12,31].

In this study, the heritability estimates of the ratio methane traits MeY and MeI were lower than those reported by Manzanilla-Pech et al. [31] using genomic information, which were 0.30 for MeY and 0.42 for MeI. However, the current estimate for MeI was identical to that reported by Lassen and Lovendahl [11] (0.21). For both MeY and MeI, the estimates found in this study were similar to those reported by van Engelen et al. [29] (0.21 and 0.18, respectively). In a meta-analysis, Brito et al. [18] reported a range of heritability estimates from 0.19 to 0.24 across all three methane traits in a total of eighteen peer-reviewed papers, including estimates from both sheep and cattle. Therefore, the estimates obtained in this study are close to or within range of those reported in ruminants and showing that methane emission traits are moderately heritable and could respond well to genetic selection.

The permanent environmental variances were large for methane traits, leading to moderate repeatabilities of 0.54 (0.03), 0.49 (0.03), and 0.74 (0.02) for MeP, MeY, and MeI, respectively (Table 3). These results suggest that all traits would benefit from being measured multiple times. Repeatability is an indicator of the correlation of two phenotypic observations taken on the same animal [32]; therefore, it represents the upper limit of a trait’s heritability [21]. The repeatability estimates found in this study are within the range of values previously reported in the literature for methane production (from 0.36 to 0.79) [5,31,33,34].

**Figure 2:**
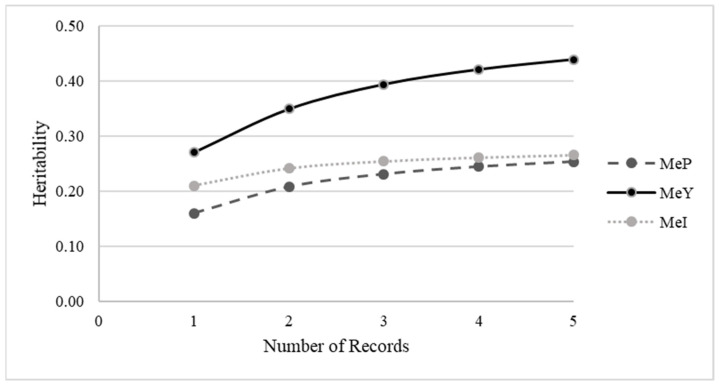
Heritability estimates for a single record and for the average of different numbers of methane emission records (*n* = from 2 to 5) for methane production (MeP), methane yield (MeY), and methane intensity (MeI).

Currently, the ideal number of days an animal’s methane emission should be measured in order to better capture its genetic merit is unknown [31], although recent studies have shown that methane emission, like feed efficiency, is dynamic and changes over the lactation (e.g., [28]). The expected heritability of the average methane production traits using one to five records recorded between 120 and 150 days in milk was estimated, and is presented in Figure 2. Within this timeframe, and as expected based on the estimated repeatabilities, our results show that MeY benefits considerably from an increased number of measurement days, with heritability estimates increasing from 0.27 to 0.44, while MeI and MeP estimates increased from 0.21 to 0.27 and from 0.16 to 0.25, respectively. From these results, one could conclude that MeI and MeP estimates during this time frame would not benefit from additional measurements after five days, considering that their response curve flattens after three measurement days. In contrast, MeY would benefit from an increased number of measurements. The higher response of MeY with increased records may be explained by the fact that MeY is a ratio trait that accounts for dry matter intake, which shows high daily variability [35,36,37,38].

Heritability estimates for dry matter intake have been shown to vary throughout lactation [39,40,41]. The same pattern has been observed with methane emissions, where heritability estimates fluctuate throughout the lactation [28]. In the current study, cows were measured for methane emissions in mid-lactation (120 to 150 DIM) for a period of five consecutive days as part of a larger project that aims to increase feed efficiency after the peak of lactation, i.e., after 60 DIM [13,42]. Additionally, it is worth noting that despite repeated measures having added value, if the tests are separated by longer periods (i.e., days to weeks) their repeatability could be higher compared to shorter term measurements (i.e., minutes to few hours). This is likely due to differences in the duration and volume of feed consumed prior to methane emission testing [43]. Thus, more measures (between three and twenty) over an extended period of time could be taken to maximize heritability [43]. While this requires further investigation, it could be less practical and feasible when considering application in breeding programs. It was recently shown that averaging the methane measurements per week can result in higher heritability and repeatability and lower residual variance of estimates [44].

### 3.4. Correlations

Phenotypic and genetic correlations are presented in Table 4. Genetic correlations (SE) between MeP and the ratio methane traits MeY and MeI were estimated to be 0.73 (±0.26) and 0.94 (±0.23), respectively. Estimated genetic correlation between the ratio methane traits of MeY and MeI was 0.68 (±0.23). The estimated genetic correlations had large standard errors, likely due to the relatively small dataset used in this study.

Previous reports in the literature concerning correlations between methane traits in dairy cattle are limited. However, Kandel et al. [45] reported high genetic correlations between MeI and MeP predicted from mid-infrared milk spectra (0.71), which is similar to the results obtained in this study. Donoghue et al. [46] reported a genetic correlation of 0.50 between MeP and MeY in Angus beef cattle, which is slightly lower than the one reported in this study. Additionally, Manzanilla-Pech et al. [31] investigated methane phenotypes in Angus beef cattle and found genetic correlations of 0.62 between MeP and MeY, 0.18 between MeP and MeI, and 0.86 between MeY and MeI. Similarly, van Engelen et al. [29] reported high genetic correlation (0.63) between MeP and MeY and low genetic correlation (0.20) between MeP and MeI using milk mid-infrared predicted phenotypes, with high standard errors (±0.15–0.28). However, unlike the estimate of 0.73 in this study, van Engelen et al. [29] reported a negative genetic correlation (−0.21) between MeY and MeI. This highlights the large variability in the estimated genetic correlation among methane traits, which stresses the importance of estimating these parameters in individual populations and using larger datasets.

### 3.5. Accuracy of EBV and EBV Rank Correlations

Regardless of which methane trait is included in a selection program, the accuracy of selecting top breeding candidates is important. Average accuracies were estimated for the sires with phenotyped daughters (*n* = 103) for each methane trait (Table 3). When comparing the average accuracies estimated per methane trait, differences were small and average accuracies were low for all traits (from 0.35 to 0.41); the highest accuracy was for MeY (0.41). These low accuracies could be due to the small dataset analyzed in this study. Accuracy should increase as the dataset is enlarged by including data from international partners and by the continued recording of methane emissions in Canada. Additionally, the incorporation of genomic information into genetic evaluation should increase EBV accuracy for the methane traits. Therefore, sharing genotypes and phenotypes with other countries to increase the size of the reference population seems to be a useful endeavor.

Spearman rank-order correlation coefficients were estimated to determine the degree of re-ranking that may occur due to the use of the ratio traits MeY and MeI, accounting for dry matter intake or milk yield, respectively, or using MeP (Table 5). Considering all animals, the ranking correlation ranged from 0.88 to 0.90. However, rank correlations between the EBV of sires (*n* = 103) with phenotyped daughters were much lower, the highest being between MeP and MeI at 0.67. The rank correlations between MeP and MeY and between MeY and MeI were 0.65 and 0.64, respectively, and a large re-ranking of sires was observed. This indicates that sires who may be best for one specific methane trait may not necessarily be the best for the other methane traits. That said, a moderate to high genetic correlation between the methane traits exists, allowing for indirect response to selection.

## 4. Conclusions

Methane emission is a moderately heritable trait regardless of the expression considered, i.e., methane production, yield, or intensity. The overall rank correlations between estimated breeding values for methane emission traits in this study were high. However, rank correlations for sires with phenotyped daughters were only moderate, indicating substantial sire re-ranking and impact on selection decisions. The methane emission trait of choice depends on the breeding goals of a particular evaluation program. A larger dataset is necessary in order to increase the accuracy of the estimated breeding values for methane emission traits.

## Figures and Tables

**Table 1:** Number of Holstein cows and records, mean, standard deviation (SD), minimum (Min), maximum (Max), and coefficient of variation (CV %) of methane production (MeP), methane yield (MeY), and methane intensity (MeI).

Trait	Herd	Number of Cows	Number of Records	Mean	SD	Min	Max	CV (%)
MeP (g/day)	DRTC ^2^	58	433	355.6	92.2	138	674	26
	ODRC ^3^	272	1332	498.6	99.4	224	799	20
	Total	330	1765	463.5	115.4	135	799	25
MeY (g/kg DMI ^1^)	DRTC ^2^	54	342	17.2	4.5	5.1	34.4	26
	ODRC ^3^	233	1126	25.4	5.7	11.7	75.7	22
	Total	287	1468	23.5	6.4	5.1	75.7	27
MeI (g/kg milk)	DRTC ^2^	21	176	10.6	3.7	4.1	26.2	35
	ODRC ^3^	244	1197	15.2	3.6	6.1	29.7	24
	Total	265	1373	14.6	3.9	4.1	29.7	27

^1^ DMI: dry matter intake; ^2^ DRTC: Dairy Research and Technology Centre; ^3^ ODRC: Ontario Dairy Research Centre.

**Table 2:** Mean and standard deviation (in parentheses) of days in milk (DIM, d), age of cow (Age, m), milk yield (MY, kg/d), fat yield (FY, kg/d), protein yield (PY, kg/d), energy corrected milk (ECM, kg/d), dry matter intake (DMI, kg/d), and body weight (BW, kg) for the cows in the DRTC and ODRC herds measured for methane emission.

Herd	DIM	Age	MY	FY	PY	ECM	DMI	BW
DRTC ^1^	132 (73)	24.4 (2.0)	32.65 (6.57)	1.214 (0.297)	1.037 (0.204)	30.96 (6.10)	20.44 (3.60)	259 (30)
ODRC ^2^	140 (85)	24.5 (1.6)	30.85 (5.23)	1.241 (0.222)	1.007 (0.158)	30.62 (4.65)	19.04 (5.02)	295 (28)

^1^ DRTC: Dairy Research and Technology Centre; ^2^ ODRC: Ontario Dairy Research Centre.

**Table 3:** Estimated additive genetic ($\sigma_{a}^{2}$), permanent environmental ($\sigma_{pe}^{2}$) and residual ($\sigma_{e}^{2}$) variances, heritabilities (*h*^2^), repeatability (*r*), and average estimated breeding value accuracy for bulls with daughters for methane production (MeP), methane yield (MeY), and methane intensity (MeI).

Trait	$\sigma_{a}^{2}$	$\sigma_{pe}^{2}$	$\sigma_{e}^{2}$	*h*^2^ (SE)	*r* (SE)	Average EBVAccuracy
MeP ^1^	1147.3	2735.6	3279.6	0.16 (0.10)	0.54 (0.03)	0.36
MeY ^2^	6.5	5.1	12.3	0.27 (0.12)	0.49 (0.03)	0.44
MeI ^3^	2.5	6.2	3.1	0.21 (0.14)	0.74 (0.02)	0.32

^1^ Methane Production in g/day; ^2^ Methane Yield in g/kg dry matter intake (DMI); ^3^ Methane Intensity in g/kg milk.

**Table 4:** Estimated additive genetic (above) and phenotypic (below) correlations with their corresponding standard errors (SE) between pairs of methane traits, i.e., methane production (MeP), methane yield (MeY), and methane intensity (MeI).

Trait	MeP	MeY	MeI
MeP	-	0.73 (0.26)	0.94 (0.23)
MeY	0.67 (0.02)	-	0.68 (0.23)
MeI	0.70 (0.03)	0.63 (0.03)	-

**Table 5:** Estimated EBV rank correlation for sires with daughters (above diagonal) and all animals (below diagonal) between pairs of methane traits, i.e., methane production (MeP), methane yield (MeY), and methane intensity (MeI).

Trait	MeP	MeY	MeI
MeP	-	0.65	0.67
MeY	0.90	-	0.64
MeI	0.88	0.88	-

## Data Availability

Data used in this study were part of the EDGP and RDGP projects’ database hosted by Lactanet Canada and are not publicly available. Data cannot be shared outside of the projects’ partners as per the Strategic Alliance Agreement.
